# An IGRT margin concept for pelvic lymph nodes in high-risk prostate cancer

**DOI:** 10.1007/s00066-017-1182-1

**Published:** 2017-07-19

**Authors:** M. Groher, P. Kopp, M. Drerup, H. Deutschmann, F. Sedlmayer, Frank Wolf

**Affiliations:** 1 Department of Radiation Oncology, St. Johanns-Spital, Paracelsus Medical University of Salzburg, Müllner Hauptstraße 48, 5020 Salzburg, Austria; 2 Department of Urology, St. Johanns-Spital, Paracelsus Medical University of Salzburg, Müllner Hauptstraße 48, 5020 Salzburg, Austria

**Keywords:** Prostate, IGRT, Margins, Lymph node irradiation, Gold-mark﻿e﻿r, Prostata, IGRT, Sicherheitsränder, Lymphknotenbestrahlung, Goldmar﻿ker

## Abstract

**Purpose:**

Gold-marker-based image-guided radiation therapy (IGRT) of the prostate allows to correct for inter- and intrafraction motion and therefore to safely reduce margins for the prostate planning target volume (PTV). However, pelvic PTVs, when coadministered in a single plan (registered to gold markers [GM]), require reassessment of the margin concept since prostate movement is independent from the pelvic bony anatomy to which the lymphatics are usually referenced to.

**Methods:**

We have therefore revisited prostate translational movement relative to the bony anatomy to obtain adequate margins for the pelvic PTVs compensating mismatch resulting from referencing pelvic target volumes to GMs in the prostate. Prostate movement was analyzed in a set of 28 patients (25 fractions each, totaling in 684 fractions) and the required margins calculated for the pelvic PTVs according to Van Herk’s margin formula $M=2.5\Upsigma +1.64\left (\sigma^{\prime}-\sigma _{p}\right )$.

**Results:**

The overall mean prostate movement relative to bony anatomy was 0.9 ± 3.1, 0.6 ± 3.4, and 0.0 ± 0.7 mm in anterior/posterior (A/P), inferior/superior (I/S) and left/right (L/R) direction, respectively. Calculated margins to compensate for the resulting mismatch to bony anatomy were 9/9/2 mm in A/P, I/S, and L/R direction and 10/11/6 mm if an additional residual error of 2 mm was assumed.

**Conclusion:**

GM-based IGRT for pelvic PTVs is feasible if margins are adapted accordingly. Margins could be reduced further if systematic errors which are introduced during the planning CT were eliminated.

## Zusammenfassung

**Einführung:**

Eine Goldmarker-(GM-)basierte, bildgeführte Radiotherapie der Prostata ermöglicht inter- und intrafraktionelle Bewegungen auszugleichen und somit Sicherheitsränder der Planungszielvolumina (PTV) zu minimieren. Dies gilt jedoch nicht für Zielvolumina des pelvinen Lymphabflusses, wenn diese im Rahmen eines simultan integrierten Boost-Konzepts im selben Plan verabreicht werden. Da Bewegungen der Prostata und des Lymphabflusses unabhängig voneinander sind, müssen PTV-Ränder der pelvinen Zielvolumina neu bewertet werden.

**Methoden:**

Wir haben die Relativbewegung von Prostata zur knöchernen Anatomie bestimmt, um daraus Sicherheitsränder zu generieren, die den durch Registrierung von pelvinen PTVs zu den GM resultierenden Versatz in der Prostata kompensieren. Relative Prostatabewegungen wurden bei 28 Patienten (jeweils 25 Fraktionen, 684 insgesamt) analysiert und die entsprechenden Ränder mittels der Formel nach Van Herk $M=2,5\Upsigma +1,64\left (\sigma^{\prime}-\sigma _{p}\right )$ berechnet.

**Ergebnisse:**

Die gesamte mittlere Prostatabewegung relativ zur knöchernen Anatomie betrug 0,9 ± 3,1, 0,6 ± 3,4 und 0,0 ± 0,7 mm in A/P- (anterior/posterior), I/S- (inferior/superior) und L/R-Richtung (links/rechts). Die berechneten Ränder der Lymphabflusszielvolumina zur Kompensation dieser Bewegungen waren 9/9/2 mm in A/P-, I/S- und L/R-Richtung bzw. 10/11/6 mm unter Annahme eines zusätzlichen systematischen Fehlers von 2 mm.

**Schlussfolgerung:**

Die GM-basierte IGRT mit Referenzierung pelviner Zielvolumina ist möglich, wenn die Sicherheitsränder entsprechend adaptiert werden, um den resultierenden Versatz zur knöchernen Struktur auszugleichen. Eine weitere Reduktion der Sicherheitsränder wäre möglich, wenn systematische Fehler, die während der Planungscomputertomographie entstehen, eliminiert werden.

## Introduction

Elective pelvic lymph node (pL) irradiation of high-risk prostate cancer, although discussed controversially, is common practice in many institutions and has traditionally been performed as a sequential boost regimen in normal fractionation for both prostate and pL planning target volumes (PTVs). Thus, the first plan including lymph node as well as prostate PTVs is typically registered to the bony anatomy whereas the following prostate boost PTV can be matched to the gold fiducials residing in the prostate.

With the advent of intensity-modulated radiotherapy (IMRT) and the recognition that prostate cancer might benefit from higher doses per fraction, simultaneous integrated boost concepts evolved and have proven feasible [1–4]. These newer concepts, however, require all PTVs to be administered within a single plan, implying that registration of prostate and pelvic PTVs can no longer be carried out separately. Therefore, PTV margins of pelvic PTVs need to be reassessed in order to compensate for mismatch resulting from relative movement of the prostate.

The prostate has been shown to move substantially within the pelvis due to differences in bladder and rectum filling [5, 6] and therefore moves independently from the pelvic bony anatomy to which the lymphatics are attached to [7, 8].

While the extent of intra- and interfractional prostate movement has been studied extensively [6, 9–11], data on relative movement to the bony anatomy rather than to skin markers are scarce, and margin recommendations for pelvic target volumes vary heavily [12–14].

We have therefore reassessed prostate movement relative to bony structures in a set of 28 consecutive high-risk prostate cancer patients and established margins for pL target volumes using Van Herk’s margin formula [15, 16].

## Materials and methods

### Patient preparation, contouring, and IMRT planning

Gold marker (GM) implantation, planning CT (3 mm slice thickness), and planning MRI were performed as previously described [17]. Prior to imaging, patients were instructed to have a full bladder and to empty their bowels using mild laxatives. If bladder filling was not sufficient, the CT scan was repeated after patients were given 500 ml to drink over a time period of 30 min. In addition, bladder volume was assessed using an ultrasound device (Bladderscan BVI 9400, Verathon, Inc.), and a minimum bladder volume for treatment specified by the treating physician (usually between 150 and 350 ml). Prior to each treatment fraction, bladder volume was assessed by the radiation therapists using Bladderscan^TM^ and checked for meeting the specifications. During the whole treatment period, patients were prescribed medication to reduce excess gas in the bowels (Antiflat, G.L. Pharma GmbH, Lannach). Contouring was performed using our in-house developed treatment and planning software Open RadART. Contouring of the prostate and seminal vesicles (sv) was performed on MRI images in transversal, sagittal, and coronal views. The final clinical target volumes (CTVs) were then contoured on the planning CT based on the superimposed MRI contours. pL CTVs were contoured on the planning CT according to RTOG contouring guidelines [18]. The upper border was the distal common iliac artery, approximately at the L5/S1 interspace. The lower border was the top of the symphysis pubis.

Inverse IMRT planning was performed using Raystation software v.4.7.2.5 (Raysearch Laboratories, Sweden). Treatment plans have been generated using either VMAT dual arc (91 segments each) or 13-field step and shoot delivering 67.5 Gy, 60 Gy, and 50 Gy in 25 fractions to the prostate, sv, and pL, respectively.

### Relative movement prostate—bony anatomy

IGRT was routinely carried out using two orthogonal kilovolt images, typically at 0 and 90°. The X‑ray images were acquired using Elekta XVI, v. 4.2.1 at 120 kV and 40 mAs. Contoured structures corresponding to the planning CT (such as GMs, organs at risk [OARs], and the bony anatomy) were then projected onto these planes. GM registration was performed automatically using an in-house developed algorithm [19]. Relative offsets of the actual GM positions and the planning situation were automatically transformed into executed couch translations. For our study, consecutive matching to the bony anatomy (symphysis, sacrum) was performed manually by our radiation therapists. The relative offsets of these two registrations were recorded in the anterior/posterior (A/P), inferior/superior (I/S), and left/right (L/R) directions for each fraction of each patient individually.

### Margin calculation

Margin calculation based on variations in prostate position was carried out using Van Herk’s margin formula [15, 16]: $M=2.5\Upsigma +1.64\left (\sigma^{\prime}-\sigma _{p}\right )$ where $\Upsigma$ is derived by calculating the standard deviation (SD) of the mean of the daily shifts of each patient, while $\sigma^{\prime}=\sqrt{\sigma ^{2}+\sigma _{p}^{2}}$ with $\sigma$ being the root mean square of the SD of the daily measurements of each patient (Table 1). $\sigma _{p}$which correlates with the penumbra width, was set to 5 mm^1^. This formula ensures a minimum dose to the pL CTVs of 95% for 90% of the patient population.

**Table 1:** Systematic (∑) and random (*σ*) errors for each direction

	A/P (mm)	I/S (mm)	L/R (mm)
$\Sigma$	3.1	3.4	0.7
*σ*	2.0	1.8	0.6
*M*	8.5	9.0	1.9
*M’*	10.0	10.4	5.4

*M* is the corresponding margin using Van Herk’s formula, whereas *M’* accounts for an additional systematic error $\Sigma _{S}$ = 2 mm, *A/P* anterior/posterior, *I/S *inferior/superior, *L/R* left/right

## Results

### Relative prostate movement

In all, 28 consecutive high-risk patients who had been irradiated from July 2015 to November 2016 at University Hospital of Salzburg were analyzed in terms of relative movement of the prostate in relation to the bony anatomy of the pelvis.

**Fig. 1:**
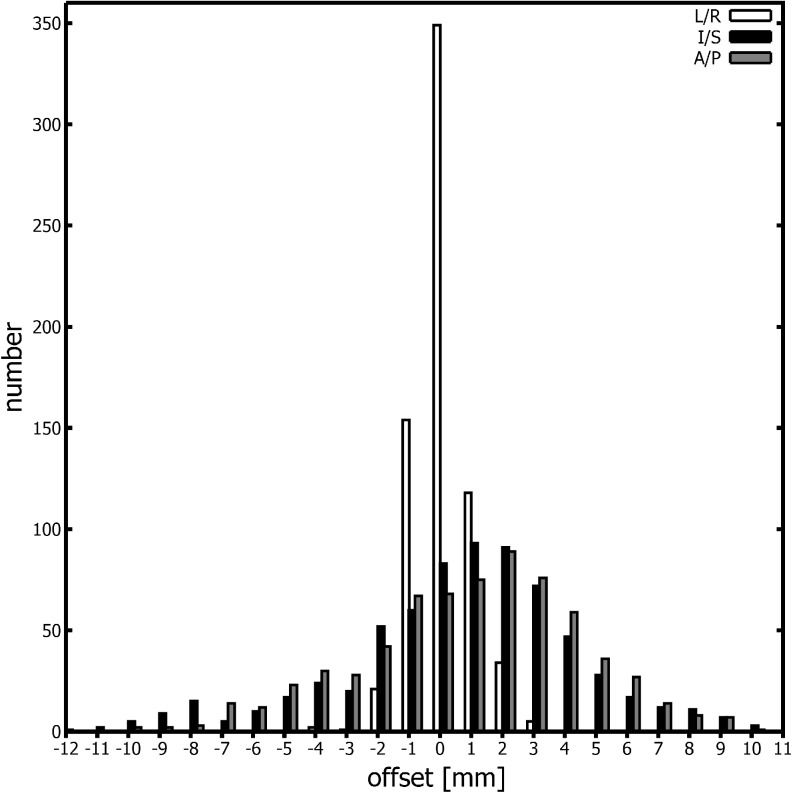
Distribution of prostate offset relative to bony anatomy for 28 patients (25 fractions each). *A/P* anterior/posterior, *I/S *inferior/superior, *L/R* left/right

In the 28 patients, a total of 684 fractions were analyzed. The overall mean prostate movement relative to bony anatomy was 0.9 ± 3.1, 0.6 ± 3.4, and 0.0 ± 0.7 mm in the A/P, I/S, and L/R directions, respectively. The distribution of prostate offsets can be seen in Fig. 1.

**Fig. 2:**
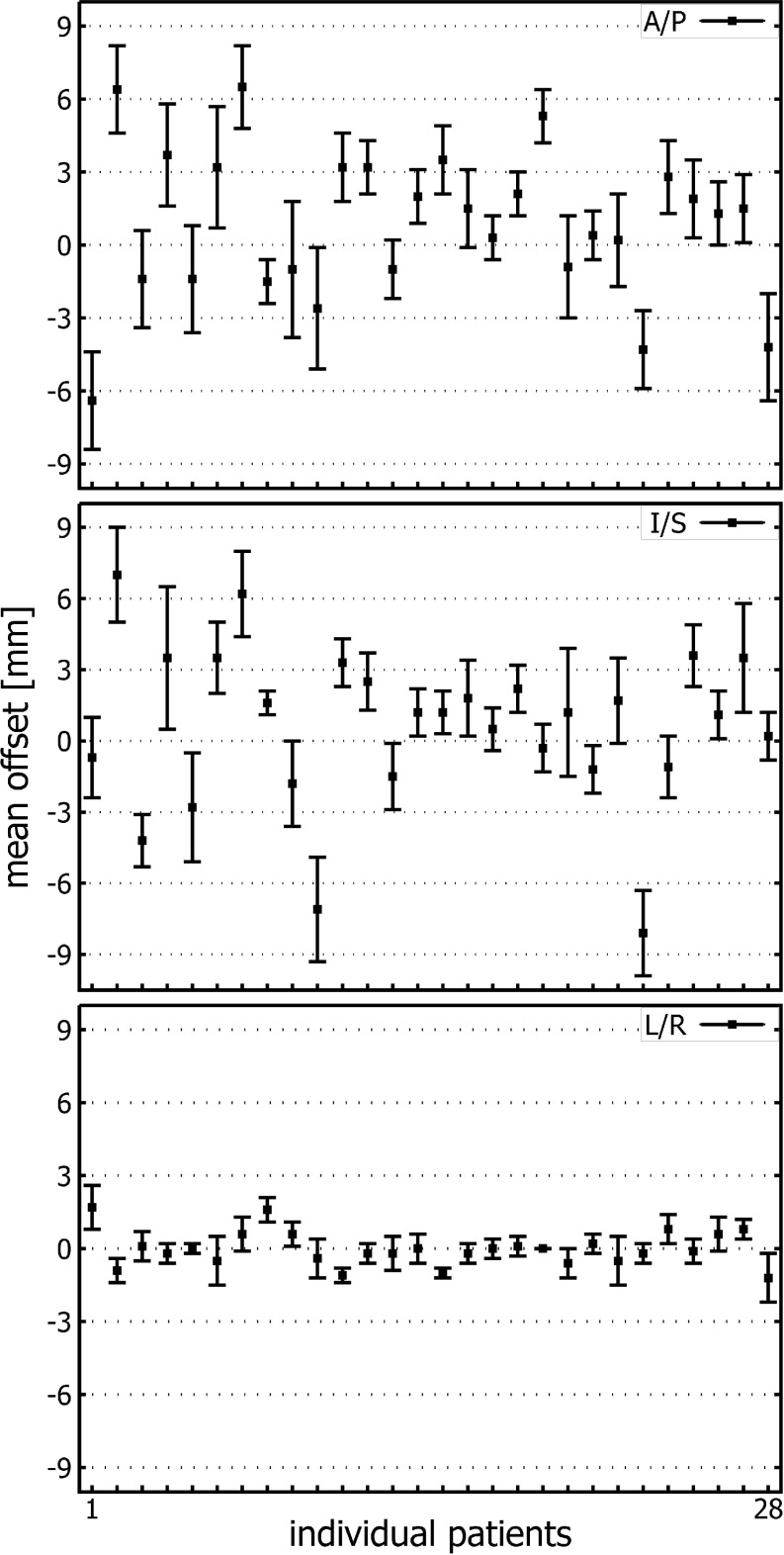
Mean offset of individual patients (1–28) in the anterior/posterior (*A/P*), inferior/superior (*I/S*) and left/right (*L/R*) directions (the error bars indicate one SD)

Interestingly, when mean prostate offset values were calculated on a patient per patient basis (Fig. 2), it became apparent that some patients exhibited larger offsets in either the I/S or A/P direction, whereas the standard deviation of offset values in patients remained low indicating little variability. The presence of large offset values (i. e., low precision) and low SD (i. e., high accuracy) indicates a systematic component of the overall error, which might have been introduced in the planning CT.

### Margins

In order to obtain a PTV expansion margin for a given CTV, a number of factors need to be determined such as delineation errors, setup errors, and organ motion. With exception of the latter, these errors need to be determined by each department individually since they are dependent on machine accuracy, contouring accuracy, and treatment time. We have therefore set those errors to zero in order to obtain a margin which exclusively compensates for errors introduced as a result from mismatch of GMs to the bony anatomy due to relative prostate motion. These minimum margins for the pL PTVs read 8.5/9.0/1.9 mm in the A/P, I/S, and L/R directions, respectively. Assuming an additional combined setup and delineation error of $\Sigma _{S}=$2 mm, the resulting margins would be 10.0/10.4/5.4 mm in the A/P, I/S, and L/R directions, respectively. We would consider these margins sufficient in the majority of modern radiation therapy units, but encourage determining the residual errors individually.

In addition, in order to quantify the influence of large systematic errors on our margins, we have identified the three patients (equivalent to about 10% of our patient population) with the largest mean displacement vector, and recalculated margins excluding them in the analysis. Resulting margins shrunk by about 1 mm in the A/P and 2 mm I/S directions and remained the same in the L/R direction (see discussion for implications of that finding).

## Discussion

In the primary external beam radiotherapy (EBRT) treatment of high-risk prostate cancer, the combination of hypofractionation to the prostate and normal fractionation to the pL is an attractive concept. GM-based IGRT and IMRT is recommended to minimize collateral damage to the rectum and bladder, but at the same time mandates to adjust PTV margins of pelvic PTVs to compensate for mismatch caused by the shift of prostate movement.

Rosewall et al. [20] have used a different approach to deal with that problem and have suggested to alternate image registration to GM and bony anatomy. However, since the prostate PTV receives a higher dose than the lymphatic PTVs, and because critical OARs such as the rectum and bladder are in close vicinity to the prostate, it makes sense to optimize PTV margins for the prostate rather than for (mostly elective) pelvic PTVs. Thus, we believe it is prudent to carry out IGRT registration of a plan that contains both prostate and lymph node PTVs, based on GMs rather than to the bony anatomy of the pelvis.

Others [12, 21, 22] have analyzed dose coverage of lymph node PTVs when image registration is performed by matching to the prostate using GM- or cone beam CT-based IGRT. However, arbitrary margins of 5–10 mm were used, and dose coverage of lymph node target volumes reported by simulating prostate offset value shifts in a small number of patients. Eminowicz et al. [22] concluded that coverage is sufficient (<0.25% coverage failure) and note in their discussion that “in an ideal setting, population based data should be used to calculate accurate CTV to PTV margins for the lymph nodes target volumes” which is exactly what we did in the present study.

We have revisited prostate movement relative to bony anatomy in a large number of 684 fractions in order to establish margins for pelvic PTV expansion which compensate for the resulting mismatch to bony anatomy when image registration is performed based on GMs in the prostate.

Our results are in good concordance with published data showing that prostate variability is very small in L/R direction and is about the same dimension in I/S and A/P direction [5, 8, 23]. This is a somewhat fortunate constellation since mediolateral PTV expansion, which contributes the most to small bowel dose, can be kept small.

It has to be noted, however, that in our margin calculation contouring errors and geometrical setup errors (such as flexmap correction inaccuracies, couch translation inaccuracies, and leaf position inaccuracies) were set to zero and need to be considered before adopting the here presented margins. For demonstration purposes, we have additionally provided margins under the assumption of a 2 mm residual error which should be adequate in most modern radiation units. However, we strongly advise to determine these errors individually in each department.

As we have shown, systematic errors, as indicated by large offsets and small SD, are introduced during the planning CT. Such systematic errors may arise through different bladder or rectum filling/distension during planning CT and treatment. In this context, routine use of laxatives prior to the planning CT but not during treatment, might be counterproductive. In addition, systematic errors could be dealt with by documenting offset values during the first three fractions and initiating replanning when a certain threshold is exceeded.

While these data demonstrate that smaller margins could be achieved if systematic errors were detected and eliminated, it also becomes clear that a rather vast effort (i. e., detection, documentation, and analysis of all offset values during the first three fractions) needs to be invested for a rather marginal gain (1 mm margin reduction in A/P and 2 mm in I/S direction).

## Conclusion

We think that GM-based IGRT for plans that contain pelvic target volumes is feasible, but margins of lymphatic PTVs need to be adapted to compensate for mismatch resulting from relative movement of the prostate. Thus, 9/9/2 mm in the A/P, I/S, and lateral directions is what we would consider minimum margins; however, larger margins might be needed to compensate for errors such as residual setup error and delineation uncertainties and need to be determined individually in each institution.

## Footnotes

1 For average static field sizes in water a lower limit for $\sigma _{p}$ of about 3.2 mm is used [16]. However, for larger fields and dynamic segments as used in our study we have derived a higher value of 5 mm.
